# Ultrastructural features mirror metabolic derangement in human endothelial cells exposed to high glucose

**DOI:** 10.1038/s41598-023-42333-5

**Published:** 2023-09-13

**Authors:** Roberta Scrimieri, Laura Locatelli, Alessandra Cazzaniga, Roberta Cazzola, Emil Malucelli, Andrea Sorrentino, Stefano Iotti, Jeanette A. Maier

**Affiliations:** 1 Department of Biomedical and Clinical Sciences, Università degli Studi di Milano, 20157 Milan, Italy; 2 Department of Pharmacy and Biotechnology, Università di Bologna, 40127 Bologna, Italy; 3 Mistral Beamline, ALBA Synchrotron Light Source, Cerdanyola del Valles, 08290 Barcelona, Spain; 4 National Institute of Biostructures and Biosystems, Viale Delle Medaglie d’Oro 305, 00136 Rome, Italy

**Keywords:** Lipids, Proteins, X-ray tomography, Molecular medicine, Cardiovascular diseases

## Abstract

High glucose-induced endothelial dysfunction is the early event that initiates diabetes-induced vascular disease. Here we employed Cryo Soft X-ray Tomography to obtain three-dimensional maps of high d-glucose-treated endothelial cells and their controls at nanometric spatial resolution. We then correlated ultrastructural differences with metabolic rewiring. While the total mitochondrial mass does not change, high d-glucose promotes mitochondrial fragmentation, as confirmed by the modulation of fission–fusion markers, and dysfunction, as demonstrated by the drop of membrane potential, the decreased oxygen consumption and the increased production of reactive oxygen species. The 3D ultrastructural analysis also indicates the accumulation of lipid droplets in cells cultured in high d-glucose. Indeed, because of the decrease of fatty acid β-oxidation induced by high d-glucose concentration, triglycerides are esterified into fatty acids and then stored into lipid droplets. We propose that the increase of lipid droplets represents an adaptive mechanism to cope with the overload of glucose and associated oxidative stress and metabolic dysregulation.

## Introduction

Due to their strategic localization, vascular endothelial cells (EC) constantly face oscillating blood glucose concentrations in relation to the pre- and post- prandial cycles^1–3^. However, uncontrolled hyperglycaemia promotes endothelial dysfunction^4^, a primary event that foreruns atherosclerosis and, therefore, cardiovascular diseases^5,6^. Physiologically, EC uptake glucose from the blood mainly through the Glucose Transporter 1 (GLUT1) and then use part of it for their own metabolism while delivering the rest to the surrounding tissues^7^. EC are highly glycolytic since most of the energy they produce derives from glycolysis^8^. Focusing on Human Umbilical Vein EC (HUVEC), the amount of glucose oxidized in the glycolytic pathway is about 200-fold higher than the glucose oxidized in the tricarboxylic acid (TCA) cycle^8^. Accordingly, mitochondrial content in EC is lower than in other cells^9^, thereby indicating that endothelial mitochondria play a role in sensing cell stress and integrating signals from the microenvironment rather than in energy production^9,10^. Mitochondria are also the headquarters of fatty acid catabolism. EC metabolize fatty acids to form Acetyl-CoA^11^, used as a fuel to sustain TCA cycle in conjunction with other anaplerotic substrates derived from glucose and/or amino acids, mainly to sustain anabolic pathways and to maintain redox homeostasis through the generation of nicotinamide adenine dinucleotide phosphate hydrogen (NADPH), necessary to reduce glutathione^12,13^. Moreover, Fatty Acid β-Oxidation (FAO) contributes to the maintenance of endothelial differentiation^14^. Of interest, EC can store neutral lipids within lipid droplets, thus providing fatty acids either to be metabolized in the mitochondria or to be transported to nearby tissues^15^.

In general, the function, content and morphology of mitochondria are highly controlled and coordinated by the balance among mitochondrial biogenesis, fission, fusion, and mitophagy^9^. Mitochondrial fusion allows the distribution of metabolites, proteins and mitochondrial DNA (mtDNA) within the cell and contributes to maintain electrical and biochemical connectivity, while mitochondrial fission is fundamental for cell division, movement and elimination of damaged or senescent mitochondria^16^. Once severely dysfunctional, mitochondria are selectively eliminated through a quality control mechanism denominated mitophagy, which is often independent of the nutrient/energy signals that govern autophagy^17^. Harmful conditions, such as redox imbalance typically associated with hyperglycaemia, lead to mitochondrial damage. Mitochondrial dysfunction has been implicated in the pathophysiology of a wide range of human diseases, including diabetes and metabolic disorders^18^. In particular, mitochondrial dysfunction is characterized by the overproduction of Reactive Oxygen Species (ROS) that cannot be neutralised by the antioxidant systems, and by a loss of efficiency in the electron transport chain^19–22^. Current evidence reports that regulators of fission, i.e. Dynamin-related Protein (DRP)1, and Mitochondrial Fission Protein 1, are increased and markers of fusion, i.e. Mitofusin (MFN)-1 and -2, and the Optic Atrophy Protein (OPA)1, are decreased in diabetes-induced endothelial dysfunction^23^. Moreover, in podocytes maintained in a hyperglycaemic environment, Rho-associated Coiled-Coil Containing Protein Kinase 1 (ROCK1), a key regulator of mitochondrial dynamics, phosphorylates DRP1 inducing mitochondrial fission and overproduction of mitochondrial ROS (mtROS) with the subsequent release of cytochrome c^24^.

To investigate the links between sub-cellular structural organization and alterations of metabolism in high d-glucose-treated EC, synchrotron-based Cryo Soft X-ray Tomography (Cryo-SXT) was used to analyse the ultrastructure of vitrified HUVEC at nanometric spatial resolution^25–27^. In HUVEC cultured in high d-glucose we found an enhanced ratio of mitochondrial fission to fusion and a significant increase of lipid droplets, which are coherent with a metabolic shift.

## Results

### Morphological changes assessed by Synchrotron-based Cryo-SXT in HUVEC exposed to high d-glucose

**Figure 1:**
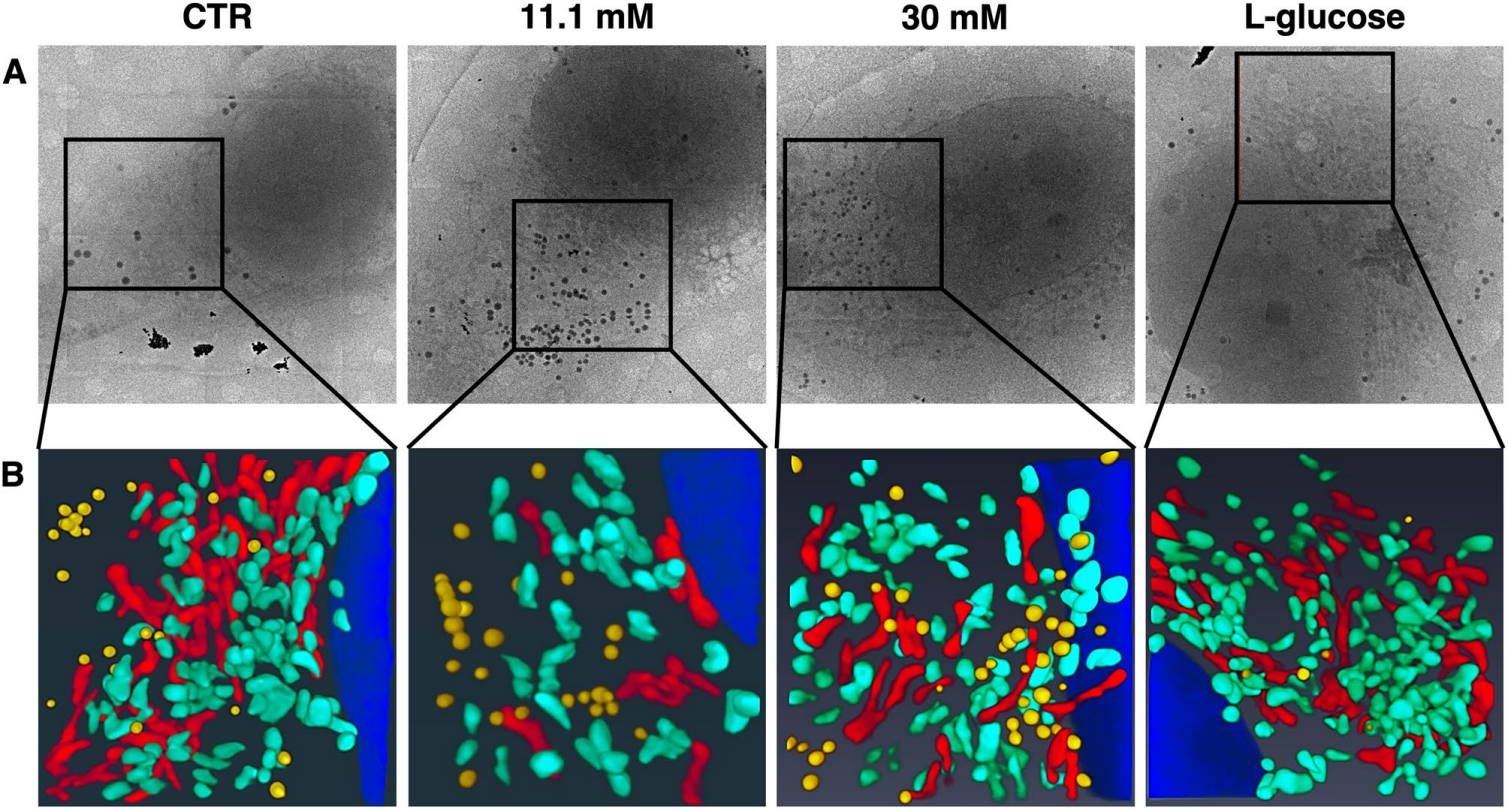
3D ultrastructural analysis of HUVEC by synchrotron-based Cryo-SXT. (**A**) 2D ultrastructure analysis of HUVEC by Cryo-SXT: it is shown one slice of the tomogram from the same volume region exposed in the panel B (pixel size 13 nm). (**B**) It is reported the corresponding color-coded manual segmentation of the selected areas of interest identifying a portion of the nucleus (blue), elongated mitochondria (red), round mitochondria (green) and lipid droplets (yellow) of 3D reconstruction.

**Figure 2:**
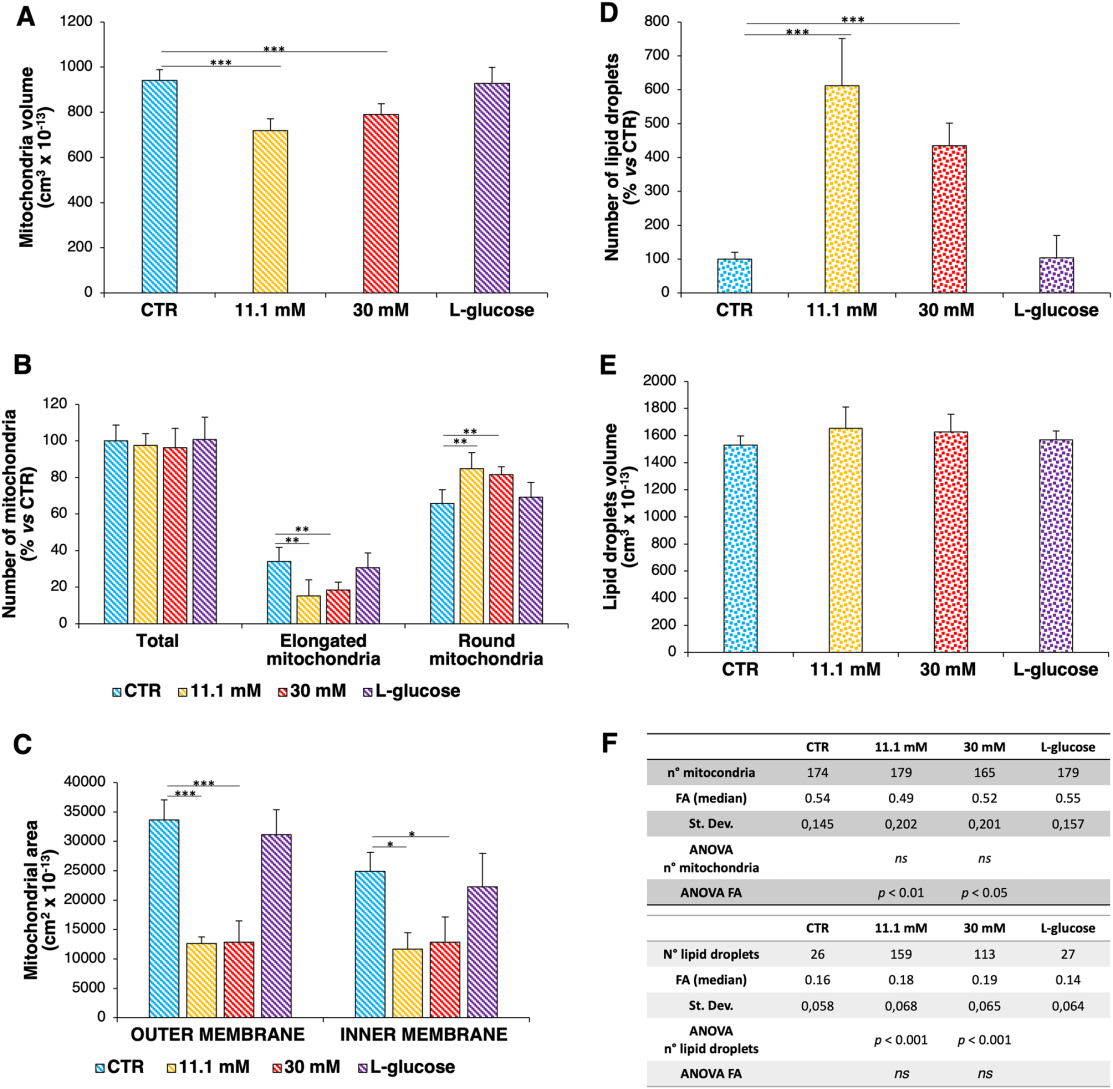
High d-glucose induces morphological alterations of mitochondria and accumulation of lipid droplets. (**A**) The graph reports the variances in the total volume of mitochondria in HUVEC cultured in physiological (CTR) or high d-glucose-containing medium. (**B**) In the graph, the total mitochondrial mass as well as the number of elongated and round mitochondria were reported, according to the different cell culture conditions. (**C**) The graph reports the variances in the areas of both the outer and inner membranes of representative mitochondria in HUVEC cultured in physiological or high d-glucose-containing medium. (**D**) In the graph, the total number of lipid droplets was reported, according to the different cell culture conditions. (**E**) The graph shows no statistically significant variances in the volume of lipid droplets in HUVEC cultured in physiological or high d-glucose-containing medium. (**F**) In the upper table, the four FA median values and the total number of mitochondria were reported. In the lower table, the four FA median values and the total number of lipid droplets were reported. The statistical analysis (11.1 mM/30 mM *vs* CTR) was calculated using one-way ANOVA. The *p*-values deriving from multiple pairwise comparisons were corrected by the Bonferroni method. In the figures, *p < 0.05, **p < 0.01, ***p < 0.001.

HUVEC were treated for 24h with physiological (5.5 mM, CTR) or high concentrations (11.1 mM and 30 mM) of d-glucose. l-glucose (30 mM) was used as control of osmolarity. Synchrotron-based Cryo-SXT was exploited to perform 3D ultrastructural quantitative analysis of mitochondria at nanoscale. The Cryo-SXT reconstructions allowed obtaining 3D nano-rendering images of the whole cells volume and to extract quantitative information about the cell compartments analysed, i.e. volume, shape and number of mitochondria and lipid droplets. After plunge freezing in liquid ethane, HUVEC were imaged in a quasi-native state, i.e. unfixed, unstained and un-sectioned. Figure 1 shows the 2D X-ray transmission images of HUVEC at different conditions and the selected region of interest analysed by Cryo-SXT (Fig. 1A). Figure 1B shows the corresponding color-coded images of the selected areas of interest obtained by the manual segmentation of the reconstructed tomographic volume. The 3D rendering of representative volume regions shows a lower number of elongated mitochondria (Fig. 1B, in red) and, in parallel, a higher number of round shaped mitochondria (Fig. 1B, in green) in HUVEC cultured in high d-glucose. Significant differences of the total volume of mitochondria in high d-glucose-cultured HUVEC *vs* their controls (CTR) were revealed (Fig. 2A), while the total mitochondrial mass is conserved (Fig. 2B). Notably, the number of elongated mitochondria, indicating fusion, is lower and the number of fragmented mitochondria, suggestive of fission, is higher in cells cultured in high d-glucose than in controls (Fig. 2B). The areas of both the outer and the inner mitochondrial membranes were significantly decreased in high d-glucose-cultured HUVEC (Fig. 2C). The 3D ultrastructure analysis of HUVEC by Cryo-SXT also highlights an accumulation of lipid droplets in high d-glucose-treated cells *vs* CTR (Fig. 1B, in yellow). The number of lipid droplets is significantly higher in the presence of high d-glucose than in control media (Fig. 2D), while no differences were detected in the dimensions of the drops (Fig. 2E). l-glucose exerts no effect.

To explore the morphological differences of mitochondria and lipid droplets in controls *vs* high d-glucose-treated HUVEC, the Fractional Anisotropy (FA) was calculated on each mitochondrion or lipid droplet segmented. FA is a scalar value, ranging between 0 and 1: when the FA verges on 0, the object is isotropic (round shape) and it is equally restricted along the three axes (λ_1_, λ_2_, λ_3_). Whereas, when FA tends to 1 the object has a preferential direction along one axis, representing the elongated shape of mitochondria. Specifically, the eigenvalues (λ_1_, λ_2_, λ_3_) allow calculating the FA as described in “Materials and methods”. The mitochondrial FA median values of HUVEC cultured in the presence of high d-glucose (11.1 mM and 30 mM) confirmed a statistically significant more isotropic shape than controls (Fig. 2F, upper table). In the lower table, we report the FA and the number of all the lipid droplets segmented for each cell culture condition (Fig. 2F).

### Mitochondrial dynamics in HUVEC exposed to high d-glucose

**Figure 3:**
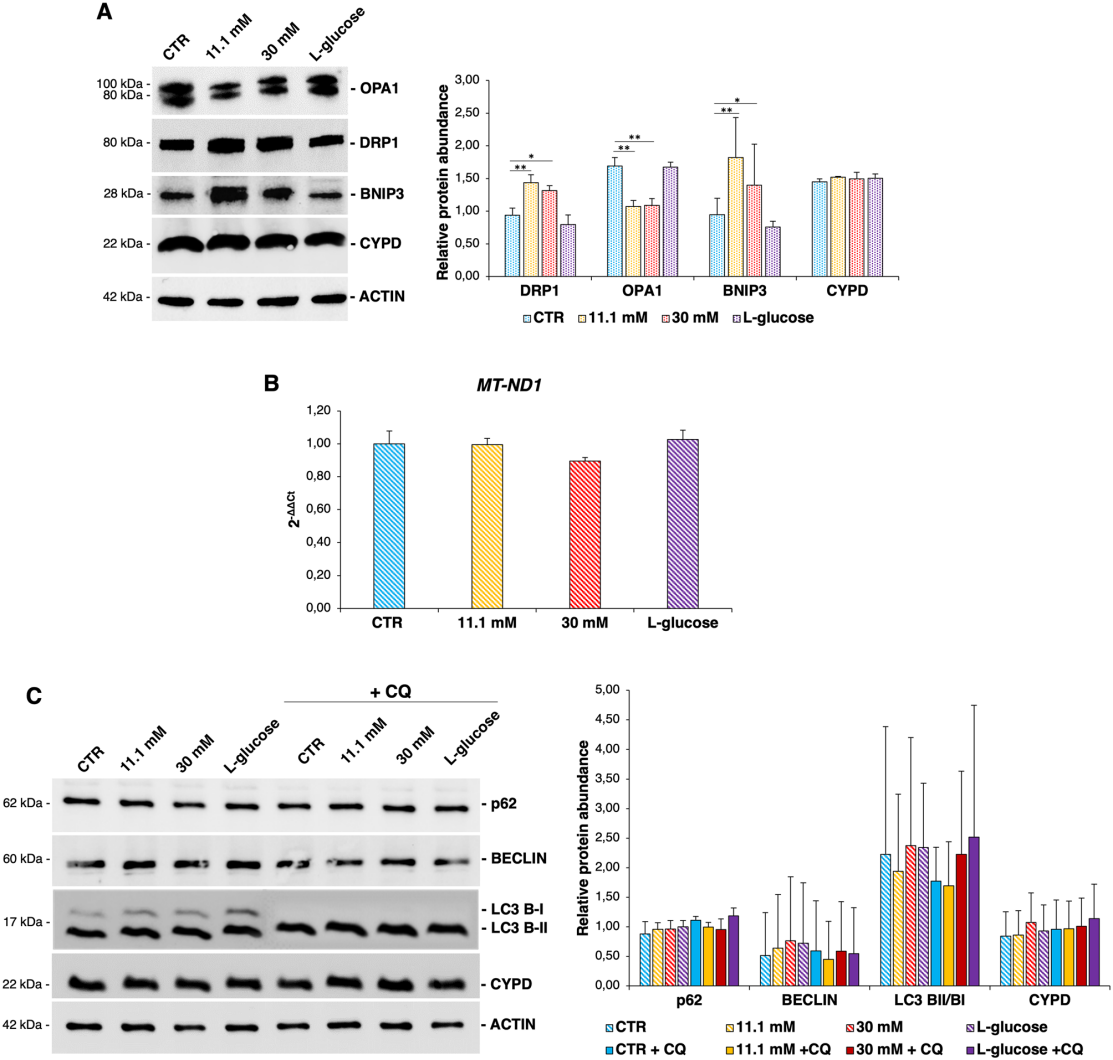
High glucose impacts mitochondrial dynamics. HUVEC were cultured in medium containing physiological or high d-glucose concentrations. (**A**) Western blot (left panel) was performed on cell lysates using specific antibodies against OPA1, DRP1, BNIP3 and CYPD. Actin was used as a marker of loading. The experiments were repeated three times and a representative blot is shown. Densitometry (right panel) was performed by Image J software calculating the ratio between the protein of interest and actin on three separate experiments ± SD. The uncropped Western blot images and the respective replicates are shown in the Supplementary data. (**B**) Real-time PCR was performed using primers designed on the *MT-ND1* sequence. *RNA45S5 *was used as internal reference gene. The experiment was repeated three times in triplicate. Values are expressed as mean ± SD and compared using one-way repeated measures ANOVA. (**C**) Western blot (left panel) was performed on cell lysates using specific antibodies against p62, BECLIN, LC3 B-I/B-II and CYPD. Actin was used as a marker of loading. The experiments were repeated three times and a representative blot is shown. Densitometry (right panel) was performed by Image J software calculating the ratio between the protein of interest and actin on three separate experiments ± SD. The uncropped Western blot images and the respective replicates are shown in the Supplementary data. In the figures, *p < 0.05, **p < 0.01.

Based on the aforementioned results, we focused on the molecular pathways regulating mitochondrial dynamics, which involve a complex signalling controlled by members of the dynamin family, among which OPA1, implicated in mitochondrial fusion, and DRP1, enrolled in mitochondrial fission. Figure 3A shows that OPA1 and DRP1 are downregulated and upregulated, respectively, in HUVEC cultured in the presence of high d-glucose. No differences emerged in cells cultured in l-glucose (30 mM), suggesting that the effect of d-glucose is not attributable to an osmotic effect. Cyclophilin D (CYPD), used as indicator of total mitochondrial content, is not modulated. That mitochondrial content does not change was confirmed by measuring the number of mitochondrial genomes per cells by Real Time PCR (Fig. 3B)^28^. Notably, the total amount of BCL2 Interacting Protein (BNIP)3, which targets mitochondria into autophagosomes thus promoting mitophagy^29,30^, is increased upon culture in high d-glucose (Fig. 3A). BNIP3 interacts directly with processed microtubule-associated proteins 1A/1B light chain 3B (LC3 B) at the autophagosome to target mitochondria to degradation^31^. Since LC3 B is cleaved when autophagy occurs, the ratio between the cleaved (LC3 B-II) and total (LC3 B-I) forms of the protein was measured and no modulation of the total amounts of LC3 B in cells exposed or not to high d-glucose was detected (Fig. 3C). We then analysed Sequestosome 1 (p62) that targets ubiquitylated proteins to the autophagosome and also regulates mitophagy^32^, and no significant differences were detected (Fig. 3C). To rule out the possibility that autophagy was too fast to show small changes in these markers, we also treated the cells with chloroquine (CQ, 40 μM), an inhibitor of autophagy that blocks the binding of autophagosomes to lysosomes by altering the acidic environment of the lysosomes. Consequently, the final expulsive phase of autophagosomes is blocked, thus allowing their accumulation and facilitating the detection of both LC3 B-II and p62^33^. No modulation of LC3 B, p62 and BECLIN, another protein involved in the regulation of autophagy, was revealed by Western blot upon CQ treatment (Fig. 3C). In parallel, no activation of autophagy was disclosed by the Tandem fluorescent-tagged LC3 B assay (data not shown). These results are in accordance with the fact that the number of mitochondria does not change, and with the lack of modulation of CYPD and mtDNA (Fig. 3A,B).

### Mitochondrial dysfunction in HUVEC exposed to high d-glucose

**Figure 4:**
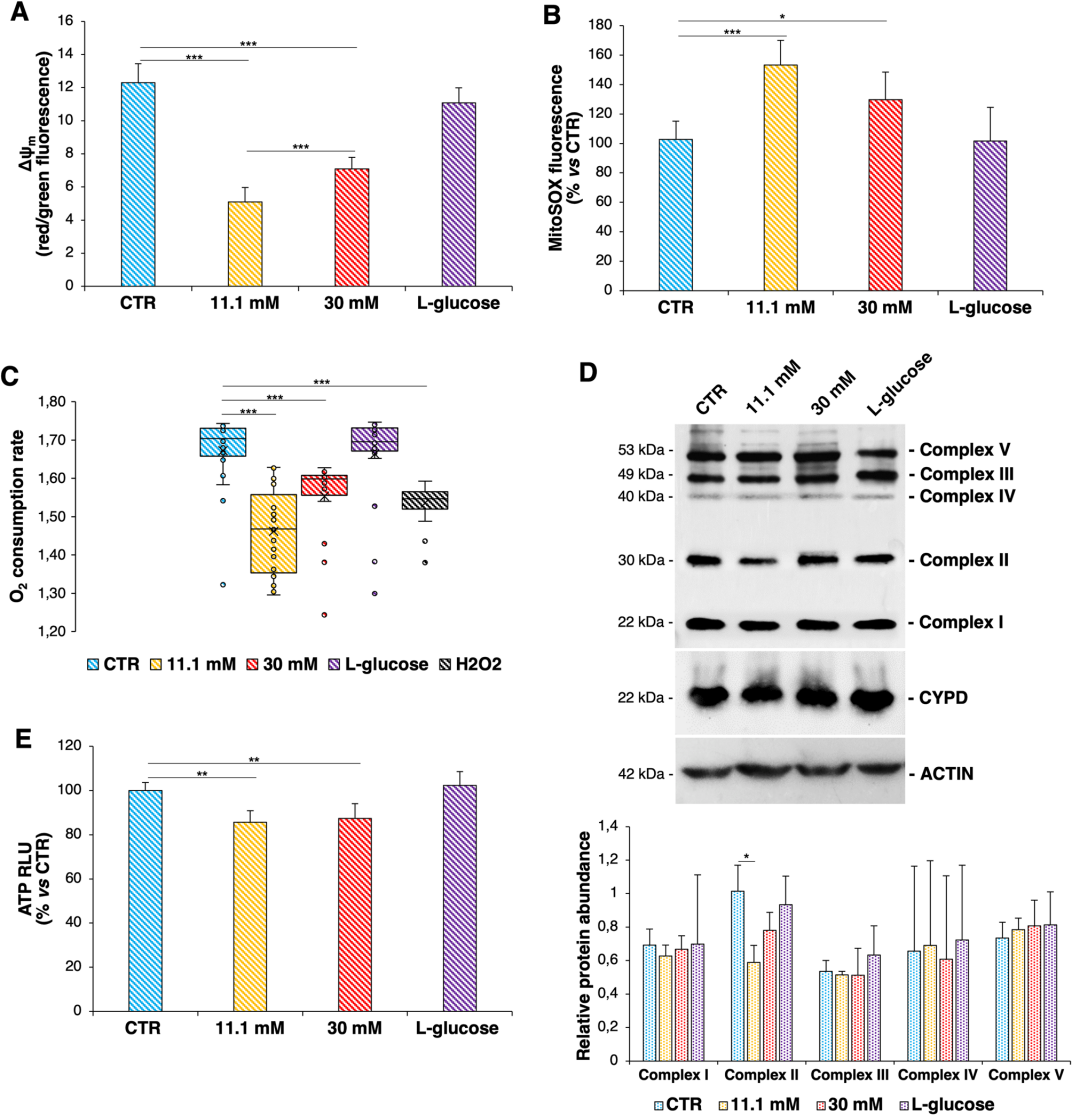
High glucose promotes mitochondrial dysfunction. (**A**) Mitochondrial membrane potential (ΔΨ_m_) was detected by JC-1 staining. Fluorescence (λ_ex/em_ red = 535/590 nm, λ_ex/em_ green 485/530) was measured using the Varioskan LUX Multimode Microplate Reader and then the red/green ratio was calculated for each sample. (**B**) mtROS production was evaluated by MitoSOX Red reagent. Values represent the means ± SD of triplicate experiments and data are shown as percentage of mtROS in HUVEC cultured in high d-glucose *vs* CTR. (**C**) The OCR was measured by Extracellular O_2_ Consumption kit as described in the Methods. H_2_O_2_ (50 μM) was used as control to mimic oxidative stress generated by high d-glucose. Values represent the means ± SD of triplicate experiments. The significance was calculated *vs* CTR. (**D**) Western blot (upper panel) was performed on cell lysates using specific antibodies against OXPHOS complexes and CYPD. Actin was used as a marker of equal loading. The experiments were repeated three times and a representative blot is shown. Densitometry (lower panel) was performed by Image J software calculating the ratio between the protein of interest and actin on three separate experiments ± SD. The uncropped Western blot images and the respective replicates are shown in the Supplementary data. (**E**) ATP was measured in isolated mitochondria. In the figures, *p < 0.05, **p < 0.01, ***p < 0.001.

Mitochondrial dysfunction is manifested by alterations of the membrane potential (ΔΨ_m_) and overproduction of ROS^34^. HUVEC were treated for 24h with physiological (5.5 mM, CTR) or high concentrations (11.1 mM and 30 mM) of d-glucose, while l-glucose (30 mM) was used as control of osmolarity. High d-glucose decreased the ΔΨ_m_ and induced the formation of mtROS (Fig. 4A,B respectively). The aberrant mitochondrial function driven by high d-glucose is also reflected by the decreased oxygen consumption rate (OCR) and the decrease in the oxidative phosphorylation Complex II (Fig. 4C,D). All these events are more pronounced in HUVEC exposed to 11.1 mM d-glucose than in cells cultured in the presence of 30 mM glucose. Mitochondrial dysfunction is also proved by the decreased production of adenosine triphosphate (ATP) in isolated mitochondria (Fig. 4E) which can be linked, in part, to the decrease of Complex II and consequently of oxidative phosphorylation and TCA cycle.

### Metabolic dysregulation in HUVEC exposed to high d-glucose

**Figure 5:**
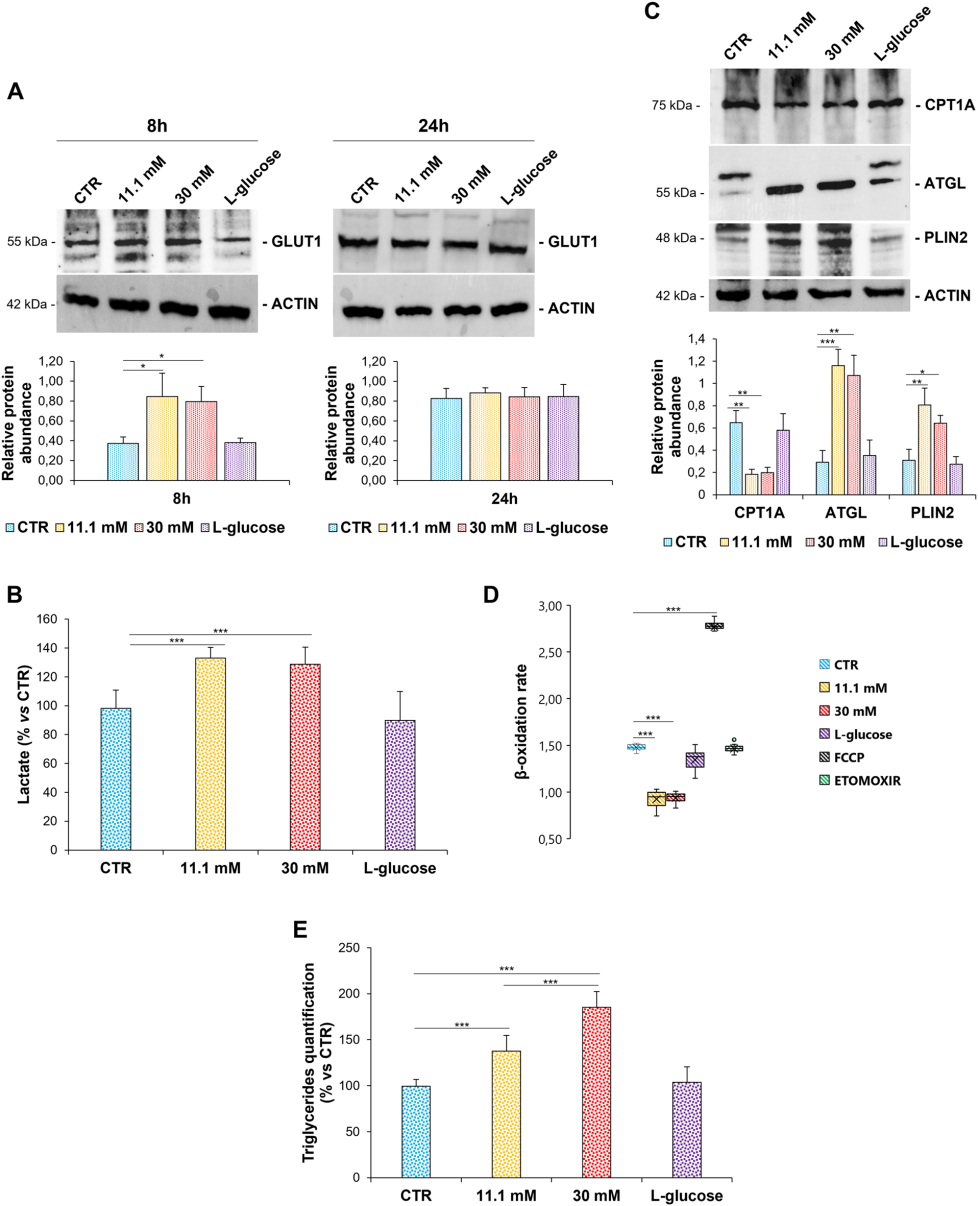
High glucose alters endothelial metabolism (**A**,**C**) Western blot (upper panel) was performed on cell lysates using specific antibodies against GLUT1, CPT1A, ATGL and PLIN2. Actin was used as a marker of loading. The experiments were repeated three times and a representative blot is shown. Densitometry (lower panel) was performed by Image J software calculating the ratio between the protein of interest and actin on three separate experiments ± SD. The uncropped Western blot images and the respective replicates are shown in the Supplementary data. (**B**) Lactate production was measured by Lactate-Glo™ Assay according to manufacturer instructions and luminescence was recorded. (**D**) The β-oxidation rate was measured using a fatty acid oxidation assay kit, as described in the methods. The FAO activator FCCP (0.625 μM) was used as the positive control, while the FAO inhibitor Etomoxir (40 µM) was used as negative control. (**E**) Triglycerides amount was detected by Triglycerides Assay Kit according to manufacturer instructions and the fluorescence was detected at λ_ex/em_ = 535/587 nm. Values are expressed as mean ± SD and compared using one-way repeated measures ANOVA. The *p*-values deriving from multiple pairwise comparisons were corrected by the Bonferroni method. The results are the mean of three experiments that were conducted in triplicate ± SD. In the figures, *p < 0.05, **p < 0.01, ***p < 0.001.

On the basis of the aforementioned results, we investigated some aspects of the metabolic response of HUVEC to high glucose. When exposed to high d-glucose, EC upregulate GLUT1 within 8h with a return to the baseline levels within 24h (Fig. 5A). Increased amounts of lactate were measured after 24h in high glucose (Fig. 5B). Moreover, high d-glucose-cultured cells markedly downregulate Carnitine Palmitoyl Transferase 1A (CPT1A) (Fig. 5C), a key enzyme in the carnitine-dependent transport of fatty acids into the mitochondria where they undergo β-oxidation. This is associated with a decreased β-oxidation rate (Fig. 5D), which might explain the accumulation of lipid droplets (Fig. 1D). Accordingly, 24 h of treatment with high d-glucose upregulates Perilipin-2 (PLIN2), a lipid droplet-associated protein whose expression mirrors the lipid content of the cells^35^ (Fig. 5C). Lipid droplets store triglycerides (Fig. 5E) in spite of the upregulation of Adipose Triglyceride Lipase (ATGL) (Fig. 5C), which catalyses the initial rate-limiting step of triglyceride hydrolysis into diglycerides and free fatty acids (FFAs).

## Discussion

The effect of high glucose on endothelial function has been the subject of many studies, with the aim to understand the initiation and progression of diabetes-induced vascular disease^1^. It is well known that cell function, shape and metabolism are interlinked^36^, however very little is known about the association between morphological changes and metabolic reshaping in EC exposed to high glucose. We therefore analysed the ultrastructure of high glucose-exposed HUVEC by Cryo-SXT and found that high d-glucose alters mitochondrial shape and promotes the accumulation of lipid droplets. The next step was to investigate mitochondrial function as well as some aspects of endothelial metabolism. In agreement with previous studies utilizing different imaging techniques^12, 37, 38^, we observed a higher number of round and a lower count of elongated mitochondria in cells cultured in high d-glucose, which indicates an imbalance between fission and fusion^39^. Accordingly, DRP1, the predominant regulator of mitochondrial fission^40^, was upregulated and OPA1, a GTPase playing an important role in fusion, was downregulated in HUVEC in high d-glucose. Interestingly, similar results were achieved in EC isolated from diabetic patients that show a lower mitochondrial network than healthy controls^41^. Increased mitochondrial fission has also been reported in retinal EC exposed to high glucose and linked to the reduction of OPA1^42^. Moreover, in high glucose treated human retinal EC and in the retinal microvasculature of human donors with documented diabetic retinopathy, mitochondrial fusion is impaired because of the hypermethylation of Mfn2 promoter^43^. Imbalances in fission and fusion seem to be a common response to high glucose as found both in vivo and in vitro. Indeed, augmented mitochondrial fission was detected in skeletal muscle cells from diabetic patients, and associated with low amounts of OPA1^44^. The downregulation of OPA1 was described also in myoblasts and pancreatic β-cells and correlated with insulin resistance^45^. In murine microvascular cells and podocytes, mitochondrial fission by high glucose was due to ROCK-mediated activation of DRP1^24^. Since an imbalance of fusion and fission not only alters mitochondrial shape but also disrupts their function^42^, it is noteworthy that HUVEC cultured in high d-glucose accumulate mtROS, which play a role in promoting mitochondrial fission^46^ together with ROS derived from cytosolic sources^47^. In fact, there are experimental pieces of evidence showing that NAD(P)H oxidases (NOX)^48^, cyclooxygenase and nitric oxide synthase^47, 49^ induce oxidative stress EC in high d-glucose, thereby contributing to endothelial dysfunction. In particular, the study by Gray et al. showed in human aortic endothelial cells exposed to high glucose an increased expression of Nox1, located in the plasma membrane^50^, accompanied by an augmentation of oxidative stress. Furthermore, within the same study, the deletion of Nox1, but not Nox4, correlated with reduced ROS formation^48^. These ROS might trigger mtROS production through a "crosstalk" between the plasma membrane and mitochondria, potentially contributing to the amplification of ROS in subcellular compartments essential for the activation of redox signalling^50^. Although it is widely accepted that mtROS production correlates positively with ΔΨ_m_, the relationships between ΔΨ_m_ and mtROS production are not fully understood yet. In fact, opposite correlations between ΔΨ_m_ and mtROS production have also been observed in some pathological conditions and mitochondrial disorders^51^. An increase in mtROS levels may be due to the overproduction and/or the decrease in enzymatic or non-enzymatic antioxidants capable of catalyzing the breakdown or scavenging of these species. Mitochondria of EC contain several potential sources of ROS associated with nutrient oxidation, such as Complex I, Complex II, Monoamine Oxidases, cytochrome c and pyruvate dehydrogenase^52^. Since we observed that the high level of d-glucose decreased the electrochemical gradient, oxygen consumption and ATP production in HUVEC, in our experimental model the main source of mtROS might be the increased activity of pyruvate dehydrogenase. Accordingly, Nishikawa et al. observed that the inhibition of glycolysis-derived pyruvate transport into mitochondria by 4-hydroxycyanocinnamic acid completely inhibited high glucose-induced ROS production in cultured bovine aortic EC^22^. In the mitochondria, the two main ROS degrading pathways are the thioredoxin-2 and glutathione systems, both requiring the reducing power of NADPH to carry out their antioxidant activities^53^. The proton gradient formed by the flow of electrons through the respiratory chain plays a fundamental role in regulating ROS levels because the return of the proton through nicotinamide nucleotide transhydrogenase is necessary for the supply of NADPH to these antioxidant systems^53^. In addition, high d-glucose induces the upregulation of the thioredoxin interacting protein (TXNIP) that inhibits the antioxidant function of thioredoxin and promotes mtROS accumulation in HUVEC^54^.

Interestingly, metformin, a mainstay of therapy in diabetic patients and particularly beneficial for the vascular system, mitigates ROS production by inhibiting DRP1-mediated mitochondrial fission in HUVEC^55^ and retards atherosclerosis in diabetic mice by ameliorating endothelial dysfunction through the reduction of mitochondrial fragmentation and the attenuation of oxidative stress^55^. We also detected a decline in mitochondrial potential, which is considered a signal of bioenergetic impairment eventually resulting in apoptosis^56^. However, in our experimental model no signs of apoptosis were observed, in agreement with previous evidence indicating that apoptosis requires longer times to be detected in high glucose-treated HUVEC^57^. As above mentioned, we also found lower amounts of ATP and decreased O_2_ consumption in high d-glucose-treated cells, in agreement with recent data in human aortic EC whose mitochondrial function was investigated by respirometry^58^. In the immortalized endothelial EA.hy926 cell line at least 6 days of culture in high glucose are necessary to detect a statistically significant decrease in basal OCR^59^. This different kinetics can be ascribed to the marked differences occurring between primary HUVEC and EA.hy926 cells^60^.

The decrease in O_2_ consumption could stem from a decrease in the O_2_ reduction reaction in water catalysed by Complex IV. This could be a consequence of the alteration in the electron transport chain due to the decreased activity of Complex II. It is noteworthy that O_2_ is also consumed by reactions catalysed by cytosolic enzymes mentioned earlier as well as by processes such as the redox cycle and lipid peroxidation. The reduction of mitochondrial oxidative processes mainly involves glutamine and fatty acids, and represents a trigger to enforce glycolysis^52^. Consequently, we investigated some aspects of lipid and glucose metabolism in HUVEC exposed to high d-glucose for 24h. We found that GLUT1 is rapidly but transiently upregulated in HUVEC in high glucose, in agreement with previous studies showing that HUVEC chronically exposed to high glucose do not modulate GLUT1^47^. To dispose the overload of glucose, the glycolytic pathway is potentiated, as evidenced by the increase of hexokinase and lactate dehydrogenase^59^. This results in the overproduction of pyruvate and, consequently, lactate along with the accumulation of glycolytic intermediates that are shunted to the polyol, pentose phosphate and hexosamine pathways, all implicated in the insurgence of endothelial dysfunction^1,47^. Among the glycolytic by-product, methylglyoxal is implicated in the vascular complications of diabetes, because it plays a role in the formation of advanced glycation end-products and the production of ROS^61^. Methylglyoxal production during glycolysis has been well documented in different kinds of cells and tissues^62^. Incubation of vascular smooth muscle cells with 25 mM glucose for 3h increases methylglyoxal production 3.5-fold and enhances oxidative stress. Moreover, 25 mM glucose and methylglyoxal induce endothelial dysfunction in rat aortic rings as well as in cultured rat aortic EC and HUVEC^63^. Therefore, it is reasonable to assume that even in our experimental model the increase in methylglyoxal may contributed to alter mitochondrial function through the inhibition of Complex II activity and decrease in Δψ_m_^64^. In the mitochondria, the conversion of excessive pyruvate into acetyl-CoA increases the production of citrate and its exportation in the cytosol as a substrate for ATP citrate lyase, which cleaves citrate to regenerate acetyl-CoA and oxaloacetate. Under conditions of glucose excess, the function of this pathway is to direct acetyl-CoA away from the mitochondria and back to the cytosol for the synthesis of fatty acids and sterols^65^. As a result, the cells can dispose glucose in excess as triglycerides stored in lipid droplets. However, an excessive increase in triglyceride synthesis can determine a high consumption of NADPH that might alter cellular redox homeostasis and, as a consequence, contribute to a decrease in the activity of the cytosolic glutathione antioxidant system resulting in endothelial dysfunction. It is relevant that lipid droplets were detected in EC lining atheromas^66,67^ and in arteries from patients bearing a loss of function mutation of ATGL^68^, a critical enzyme of triglyceride lipolysis. The significance of triglyceride-rich lipid droplets is not clear at the moment. Beyond serving as an energy resource, endothelial lipid droplets also function as a defense mechanism against lipotoxicity^67^. This mechanism might be applicable also to HUVEC in high d-glucose where ROS are overproduced. Lipid droplets also reduce mitochondrial fragmentation and ROS production^69^, as upon stress lipid droplets and mitochondria physically interact so that noxious proteins present on the outer mitochondrial membrane can be cleared^70^. In general, the accumulation of lipid droplets might be interpreted as a compensatory mechanism to dump high glucose-driven storage of triglycerides. The upregulation of ATGL in HUVEC cultured in high glucose sounds intriguing in the light of experiments showing that primary EC lacking ATGL accumulate lipid droplets. Since (i) FFAs are ligands for the lipid sensing nuclear receptor PPAR-γ, (ii) PPAR-γ upregulates ATGL^71^ and (iii) culture in high d-glucose increases the amounts of PPAR-γ^54^, we propose that ATGL upregulation is mediated through the FFA-PPAR-γ pathway. ATGL also promotes lipophagy^72^, and, in HUVEC exposed to high glucose, this might represent an initial step to prevent excessive accumulation of lipid droplets. However, in our experimental setting we did not observe any difference in autophagy, the physiological process which eliminates damaged or senescent organelles. It is possible that this process might require longer exposure times to become activated in HUVEC exposed to high d-glucose. In line with this issue, we propose that mitophagy may not play a significant role in the early remodeling of mitochondrial network in HUVEC cultured in high glucose. In this context, the upregulation of BNIP3 can be envisioned as a mechanism to regulate mitochondrial dysfunction^73^. Initially, it has a role in reducing respiration, decreasing ΔΨ_m_ and ATP synthesis, before potentially serving as a driver of mitophagy. Further studies in kinetics should be performed to test this hypothesis.

**Figure 6:**
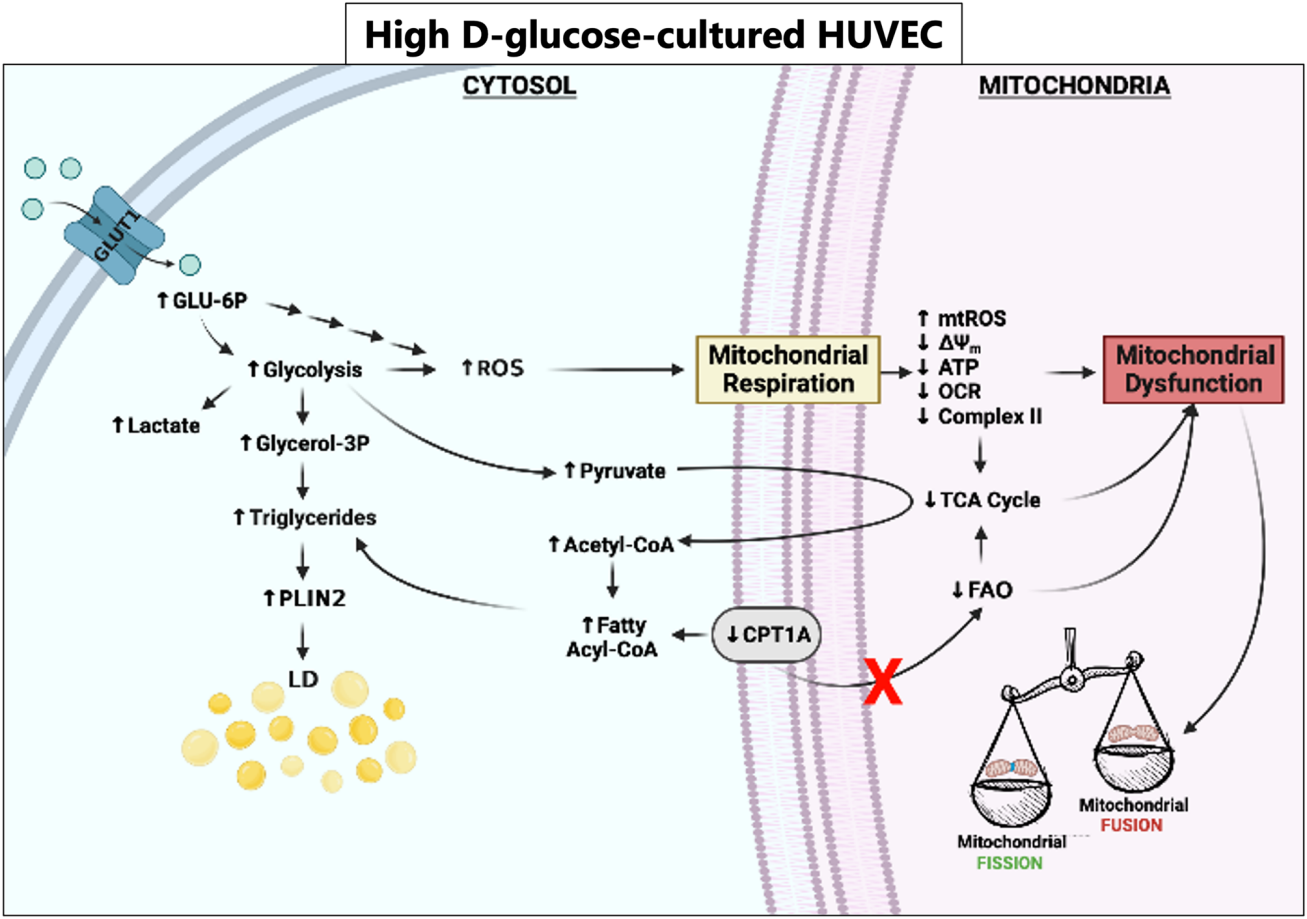
Schematic representation of the results, created in Biorender.com. GLUT1 Glucose Transporter 1, GLU-6P Glucose-6-phosphate, Glycerol-3P Glycerol-3-posphate, ROS Reactive Oxygen Species, PLIN2 Perilipin-2, LD Lipid droplets, CPT1A Carnitine Palmitoyl Transferase 1A, mtROS mitochondrial ROS, Δψ_m_ mitochondrial membrane potential, ATP adenosine triphosphate, OCR oxygen consumption rate, TCA tricarboxylic acid cycle, FAO Fatty acid β-oxidation.

Another result that caught our attention is the lack of dose dependence in some responses to high glucose. In particular, Δψ_m_ and O_2_ consumption are significantly and reproducibly lower in cells exposed to 11.1 mM than 30 mM glucose. Moreover, mtROS are higher in HUVEC treated with 11.1 mM than 30 mM glucose. We hypothesize that very high concentrations of glucose activate rapid and robust adaptive mechanisms that limit some detrimental effects of glucose overload, while lower, albeit pathological, concentrations do not grant to reach the threshold of anti-stress defense. In Fig. 6, a schematic representation of results is reported.

In conclusion, we found a relation between the ultrastructural changes of HUVEC exposed to high glucose and their metabolic derangement. More experiments are necessary for a clear definition of the time sequence of these events.

## Materials and methods

### Cell culture

HUVEC were purchased from the American Type Culture Collection (ATCC Manassas, Virginia, USA) and cultured in medium M199 supplemented with 10% fetal bovine serum, 1 mM l-Glutamine, 1 mM Penicillin–Streptomycin (Euroclone, Milano, Italy), 1 mM Sodium Pyruvate, 5 U/mL Heparin and 150 µg/mL Endothelial Cell Growth Factor on collagen-coated dishes (50 µg/mL) (Sigma-Aldrich, St. Louis, MO, USA). d-glucose was used at concentrations of 11.1 mM and 30 mM and l-glucose (Sigma-Aldrich) was utilized as control of osmolarity (30 mM). 30 mM glucose corresponds to severe hyperglycaemia in diabetic individuals and is used in many studies on EC, whereas 11.1 mM glucose is a pathological concentration rarely used in in vitro experiments.

### Sample preparation for Cryo Soft X-ray tomography (Cryo-SXT)

HUVEC were seeded onto gold Quantifoil R 2/2 holey carbon-film microscopy grids at a concentration of 1 × 10^4^ cell/cm^2^. Cells were incubated at 37 °C in 5% CO_2_ for 24h with medium containing physiological (5.5 mM, CTR) or high concentrations of d-glucose (11.1 mM and 30 mM). l-glucose was used as control of osmolarity at a concentration of 30 mM. The samples were then gently rinsed twice with phosphate buffered saline (PBS) (Euroclone) and soon thereafter HUVEC were frozen-hydrated by a rapid plunge freezing in liquid ethane bath cooled with liquid nitrogen using a Leica EM GP robot. Excess water was removed before plunge freezing via blotting to obtain a total ice thickness well below 5 µm. Frozen specimens were transferred into the full field soft X-ray transmission microscope of the beamline of the ALBA-Light Source^74^, where Cryo-SXT tomographic measurements of whole frozen hydrated cells were performed. The cryogenic conditions were maintained during all the experiment.

### Cryo soft X-ray tomography

Cryo-SXT images were recorded at the MISTRAL beamline of the ALBA light source, where photons extracted from a bending magnet source are directed on the sample by a capillary condenser facing the monochromator exit slit. Behind the sample, a zone plate with an outermost zone width of 40 nm acts as the objective lens of the microscope, generating a magnified image of the sample on a direct illumination CCD detector^74^. Cryo-SXT was carried out at 520 eV to optimize the contrast between the carbon-rich organelles membranes and the water-rich cytoplasmic solutions. For each cell, a tilt series was acquired using an angular step of 1° on a 140° angular range. The effective pixel size in the images was 13 nm. No radiation damage was detected at our spatial resolution. Each transmission projection image of the tilt series was normalized using flat-field (incident intensity) images of 1-s acquisition time. The tilt series were manually aligned using eTomo in the IMOD tomography software suite^75^. With the aim to decrease as much as possible the deviations from an ideal rotation that creates artefacts in the reconstructed tomograms, the rotation of Au fiducials of 150 nm diameter (BBI Solutions—Freiburg, Germany) was followed. According with the Beer–Lambert law, the transmission T(x,y) is given by:

$$T\left(x,y\right)=\frac{I\left(x,y\right)}{{I}_{0}\left(x,y\right)}={e}^{(-\int \mu (x,y,{E}_{0})dt)}={e}^{(-\int {\mu }_{m}(x,y,{E}_{0})\rho d{t}_{m})}$$

where I is the transmitted intensity by the sample; I_0_ is the incident beam intensity; µ is the X-ray linear absorption coefficient (LAC) at incident energy E_0_, µ_m_(E_0_) = µ/ρ is the mass absorption coefficient at the same energy; ρ is the matrix density; x and y are the coordinates in the transversal plane at the sample position and the integral is extended through all the sample thickness. All the transmission tilt series have been converted in absorbance A using ImageJ by applying the following expression:

$$A=\mu \left({E}_{0}\right)t=-ln\left(T\right)$$

The absorbance tilt series were finally reconstructed with TomoJ^76^, a plugin of ImageJ (National Institute of Health, Bethesda, MD, USA)^77^, using the ART iterative-algorithms with 15 iterations and a relaxation coefficient of 0.01. Finally, the images were segmented by Amira (Thermo Fisher Scientific, Waltham, MA, USA) and the “Volren” module enables to render the segmented regions at the same time with different colormaps.

### Fractional anisotropy

The Fractional Anisotropy (FA) was calculated for all segmented mitochondria and lipid droplets in every cell culture condition. The eigenvalues λ_1_, λ_2_ and λ_3_ were automatically extracted by Amira software and the FA has been calculated by implementing the following formula^78^:

$$FA=\sqrt{\frac{3}{2}} \frac{\sqrt{\left({\lambda }_{1}-\lambda \right)+\left({\lambda }_{2}-\lambda \right)+\left({\lambda }_{3}-\lambda \right)}}{\sqrt{{\lambda }_{1}^{2}+{\lambda }_{2}^{2}+{\lambda }_{3}^{2}}}$$

### Western blot

HUVEC were lysed in 50 mM Tris–HCl (pH 7.4) containing 150 mM NaCl (Sigma-Aldrich), 1% NP40 (Sigma-Aldrich), 0.25% sodium deoxycholate (Sigma-Aldrich), protease inhibitors (10 µg/mL Leupeptin, 10 µg/mL Aprotinin and 1 mM Phenylmethyl-Sulfonyl Fluoride, PMSF) (Sigma-Aldrich), and phosphatase inhibitors (1 mM sodium fluoride, 1 mM sodium vanadate, 5 mM sodium phosphate) (Sigma-Aldrich). Lysates (40 µg/lane) were separated by SDS-PAGE and transferred to nitrocellulose sheets. Western Blot analysis was performed using antibodies against OPA1, DRP1, LC3 B-I/-II, BECLIN (Cell Signalling, Euroclone, Pero, Italy), CYPD, CPT1A, GLUT1 (Thermo Fisher Scientific), BNIP3 (Sigma-Aldrich), p62 (Invitrogen, Carlsbad, CA, USA), ATGL, PLIN2 and mitochondrial oxidative phosphorylation complexes (OXPHOS) (Abcam, Cambridge, UK). Actin (Santa Cruz, Dallas, Texas, USA) was the control of equal loading. After washing, secondary antibodies labelled with horseradish peroxidase (GE Healthcare, Waukesha, WI, USA) were used. Immunoreactive proteins were detected with Clarity™ Western ECL substrate by ChemiDoc MP Imaging System (Bio-Rad). Densitometry of the bands was performed with ImageJ. The Western blots shown are representative and the densitometric analysis was performed calculating the ratio between the protein of interest and actin on three independent experiments ± Standard Deviation (SD).

### Real-time PCR

Real-time PCR (RT-PCR) was performed three times in triplicate using the CFX96 Touch Real-Time PCR Detection System (Bio-Rad) exploiting the TaqMan Gene Expression Assay (Life Technologies, Monza, Italy). The following primers were used: Hs02596873_s1 (MT-ND1) and Hs03654441_s1 (RNA45S5) as internal reference gene. Relative changes in gene expression were analysed by the 2^−ΔΔCt^ method.

### Mitochondrial membrane potential (ΔΨ_m_) and mtROS production

ΔΨ_m_ was quantified by measuring fluorescence intensities of red-shifted aggregates (in functional mitochondria) and green-shifted JC-1 (in damaged mitochondria) monomers to evaluate mitochondrial viability using JC-1 probe (Thermo Fisher Scientific). The cells were incubated with the probe at 37 °C for 10 min, and fluorescence (λ_ex/em_ red = 575/590 nm, λ_ex/em_ green = 460/510 nm) was measured using the Varioskan LUX Multimode Microplate Reader (Thermo Fisher Scientific). The red/green ratio was calculated for each sample^79^.

mtROS production was measured by MitoSOX™ Red mitochondrial superoxide indicator (Invitrogen). After the treatments in a 96-well plate, the cells were incubated for 10 min at 37 °C with the reagent, protected from light. Fluorescence was measured at λ_ex/em_ = 510/580 nm using the Varioskan LUX Multimode Microplate Reader.

### Extracellular O_2_ consumption

The Oxygen Consumption Rate (OCR) was measured by the Extracellular O_2_ Consumption Reagent (Abcam), according to the manufacturer’s instructions. In particular, the assay is based on the ability of oxygen to quench the excited state of the reagent. During the cell respiration, the oxygen is depleted in the surrounding environment increasing the fluorescent signal. After the treatments, the cells were incubated with the Extracellular O_2_ Consumption Reagent and each well was sealed by adding pre-warmed High Sensitivity mineral oil^80^. H_2_O_2_ was used as positive control. Then the plate was introduced into the Varioskan LUX Multimode Microplate Reader (Thermo Fisher Scientific), pre-set to 37 °C. The fluorescent signal was measured every 2 min for 180 min at λ_ex/em_ = 380/650 nm and normalized on the cell number. The results are the mean of three independent experiments ± SD.

### ATP quantification

The CellTiter-Glo Luminescent Cell Viability Assay (Promega, Madison, Wisconsin, USA) was used to determine the quantification of the mitochondrial ATP, according to the manufacturer’s instructions. This assay relies on the properties of a thermostable luciferase that, in the presence of Mg^2+^, catalyses an oxidative reaction thus producing bioluminescence. Starting from luciferin and the co-factors molecular oxygen and ATP, it is produced oxyluciferin and emitted light. After the treatments, ATP content was measured on permeabilized mitochondria. Thus, the cells were trypsinized, permeabilized, resuspended in isolation buffer (100 mM KCl, 50 mM TRIS, 5 mM MgCl_2_, 1.8 mM ATP, 1 mM EDTA), and centrifuged at 600*g* for 10 min at 4 °C. The supernatant was centrifuged at 10,000*g* for 15 min at 4 °C to allow the sedimentation of mitochondria, which were incubated in CellTiter-Glo Reagent, diluted in culture medium with a 1:1 ratio, for 10 min at room temperature. The luciferase activity was monitored using the Varioskan LUX Multimode Microplate Reader (Thermo Fisher Scientific). The fluorescent results were normalized on the cell number. The results are the mean of three independent experiments performed in triplicate ± SD.

### FAO and triglycerides quantification

FAO was monitored by Fatty Acid Oxidation assay (Abcam) in living cells seeded in a 96-well black plate (Greiner Bio-One). Oleate was the substrate utilized to measure fatty acid-driven oxygen consumption^54,81^. Cells were treated with high glucose for 24h and, at the end of the experiment, were rinsed twice with 90 μL of pre-warmed FA-Free Measurement Media (containing 150 μM oleate-BSA conjugate) and then added 10 μL of extracellular O_2_ consumption reagent. Extracellular O_2_ Consumption Reagent (Abcam) was added into all the wells except for the blank control well. Carbonyl cyanide-p-trifluoromethoxyphenylhydrazone (FCCP, 0.625 µM), used as positive control, induces maximal electron transport chain activity by dissipating the mitochondrial membrane potential. Etomoxir (40 µM), an inhibitor of the carnitine transporter CPT1, was used as negative control. At the end of the experiment, the wells were sealed with pre-warmed high sensitivity mineral oil. The Varioskan LUX Multimode Microplate Reader (Thermo Fisher Scientific) was used (λ_ex/em_ = 380/650 nm) was measured every 2 min for 180 min^54,81^.

Triglycerides were quantified using Triglyceride Quantification Kit (Sigma-Aldrich), according to the manufacturer’s recommendations. Triglycerides are hydrolyzed by lipoprotein lipase to glycerol and free fatty acids. Glycerol is then measured by coupled enzyme reactions resulting in the final production of a quinoneimine dye that shows an absorbance at 540 nm. The increase in absorbance at 540 nm is directly proportional to triglyceride concentration of the sample. Fluorescence (λ_ex/em_ = 535–587 nm) was monitored using the Varioskan LUX Multimode Microplate Reader (Thermo Fisher Scientific). The fluorescent results were normalized on the cell number^54^.

The results are the mean of three independent experiments performed in triplicate ± SD.

### Lactate quantification

L-Lactate was quantified using the luminescence-based Lactate-Glo™ Assay (Promega), according to the manufacturer’s recommendations. In the presence of NADH, a pro-luciferin reductase substrate is converted to luciferin by reductase, emitting light proportionally to the amount of lactate in the sample. Luminescence was monitored using the Varioskan LUX Multimode Microplate Reader. The results were normalized on the cell number. The results are the mean of three independent experiments performed in triplicate ± SD.

### Statistical analysis

All the results are the mean of three independent experiments performed in triplicate ± SD. The data were analysed using one-way ANOVA. The *p*-values deriving from multiple pairwise comparisons were corrected using the Bonferroni method. The statistical analysis was performed with the software GraphPad Prism. Statistical significance was defined as *p*-value < 0.05. Regarding the figures, *p < 0.05; **p < 0.01; ***p < 0.001.

### Supplementary Information

Supplementary Figures.

## Data Availability

The data presented in this study are openly available in Dataverse at the following link: https://doi.org/10.13130/RD_UNIMI/P47RRZ.
