# Supplementary material for: Ultrastructural features mirror metabolic derangement in human endothelial cells exposed to high glucose
Source: Sci Rep. 2023 Sep 13;13:15133. doi: 10.1038/s41598-023-42333-5 (PMC10499809; doi:10.1038/s41598-023-42333-5)
Supplement: Supplementary file 1 — Supplementary Figures. [file 41598_2023_42333_MOESM1_ESM.docx]

**ULTRASTRUCTURAL FEATURES MIRROR METABOLIC DERANGEMENT IN HUMAN ENDOTHELIAL CELLS EXPOSED TO HIGH GLUCOSE**

Roberta Scrimieri ^1*^, Laura Locatelli ^1^, Alessandra Cazzaniga ^1^, Roberta Cazzola ^1^, Emil Malucelli ^2^, Andrea Sorrentino ^3^, Stefano Iotti ^2,4^, Jeanette A. Maier ^1*^

**SUPPLEMENTARY DATA**


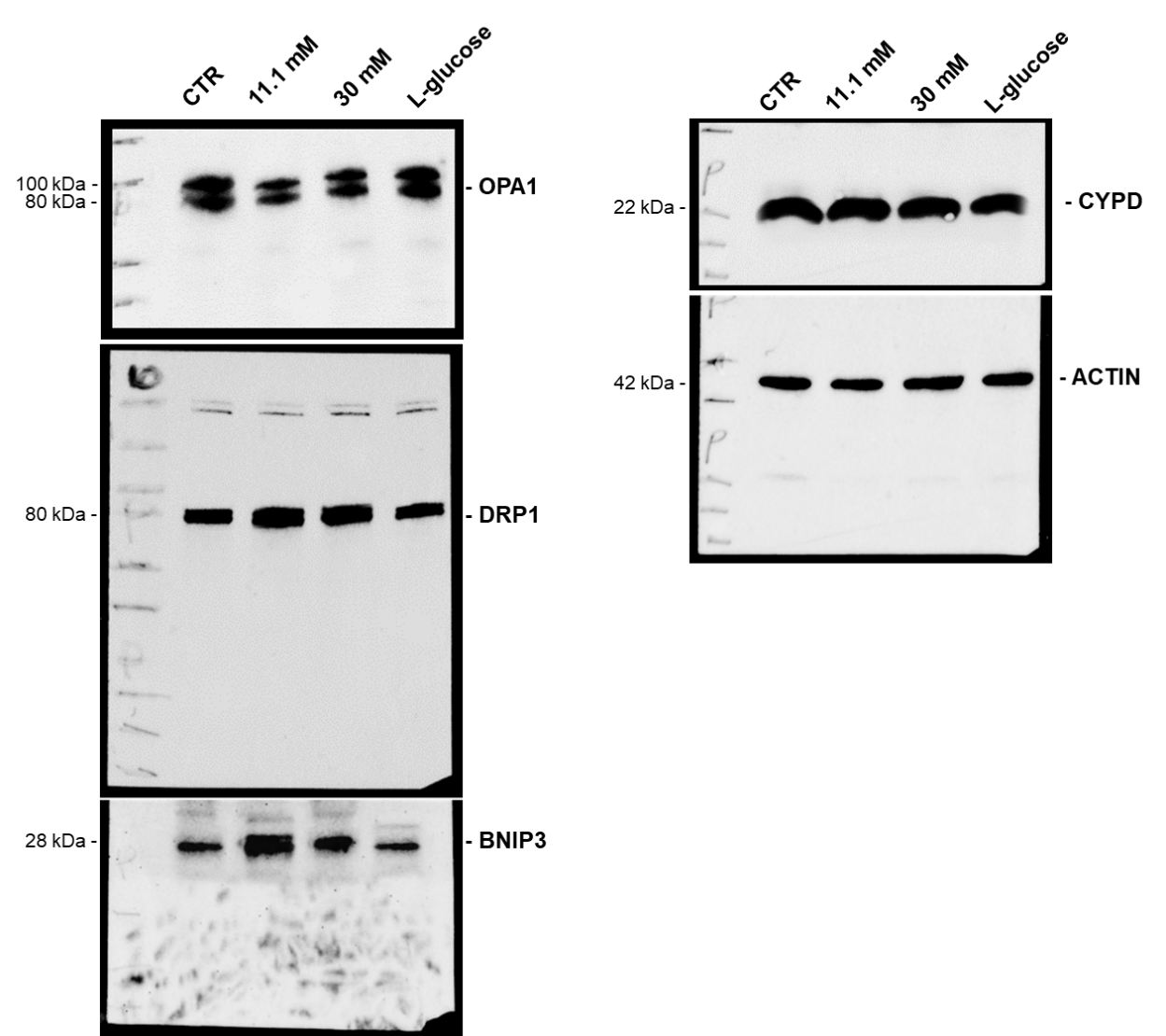
**FIGURE 3A – UNCROPPED:**

**Figure 3A – Uncropped**. Images of original uncropped Western blots used for preparation of Figure 3A. Western blot was performed on cell lysates using specific antibodies against OPA1, DRP1, BNIP3 and CYPD. Actin was used as a marker of loading.

**
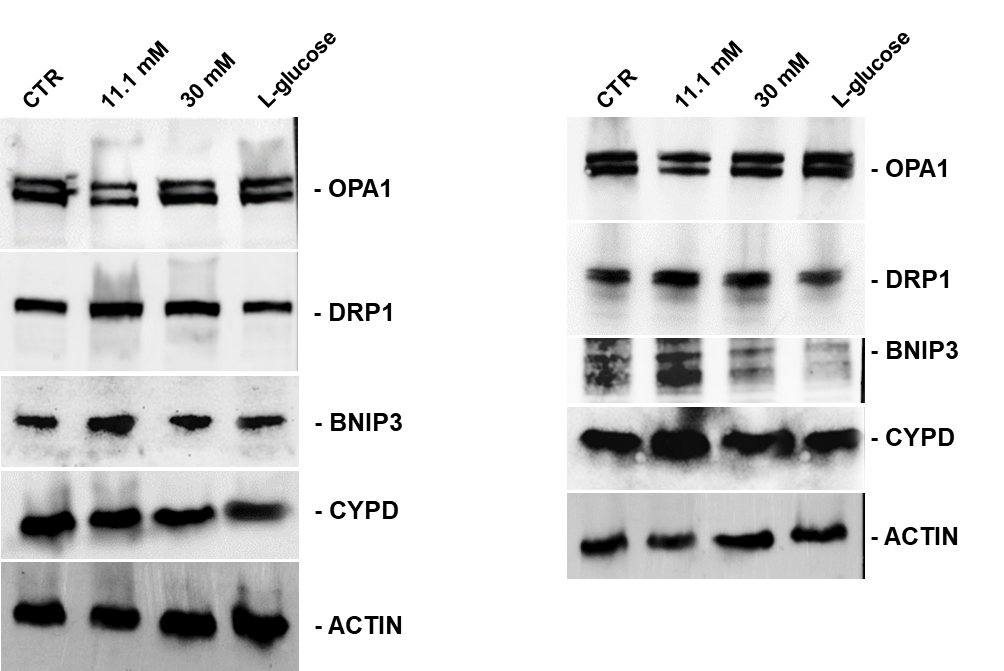
FIGURE 3A – REPLICATES:**

**Figure 3A – Replicates**. Western blot replicates of Figure 3A.


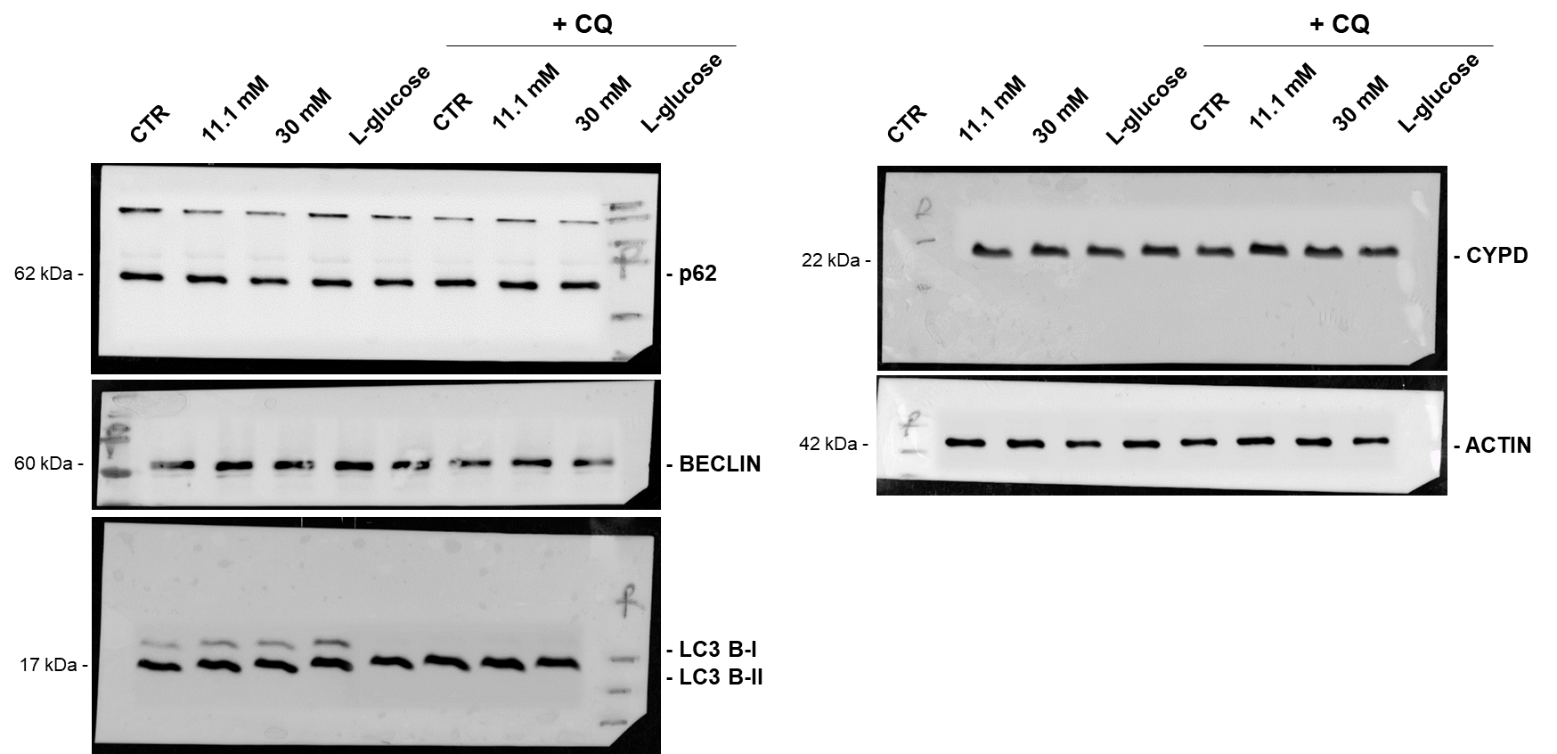
**FIGURE 3C – UNCROPPED:**

**Figure 3C – Uncropped**. Images of original uncropped Western blots used for preparation of Figure 3C. Western blot was performed on cell lysates using specific antibodies against p62, BECLIN, LC3 B-I/B-II and CYPD. Actin was used as a marker of loading.


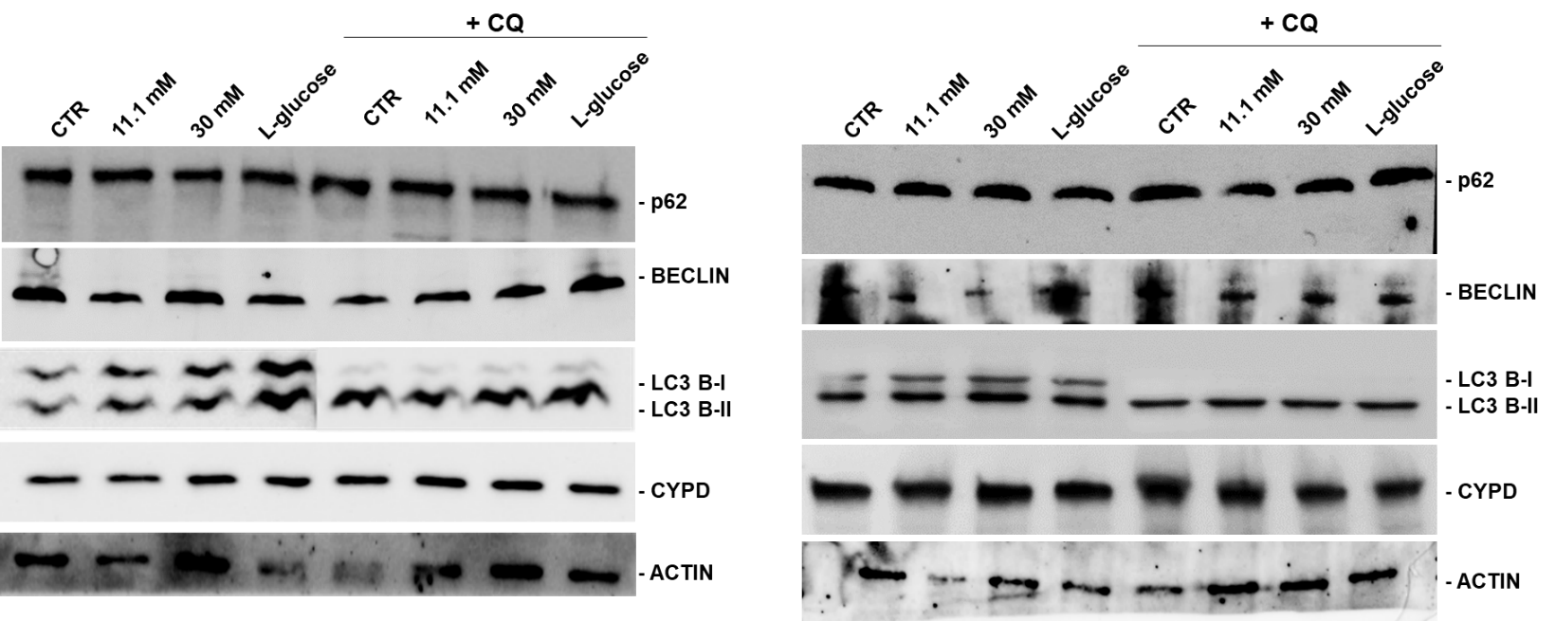
**FIGURE 3C – REPLICATES:**

**Figure 3C – Replicates**. Western blot replicates of Figure 3C.


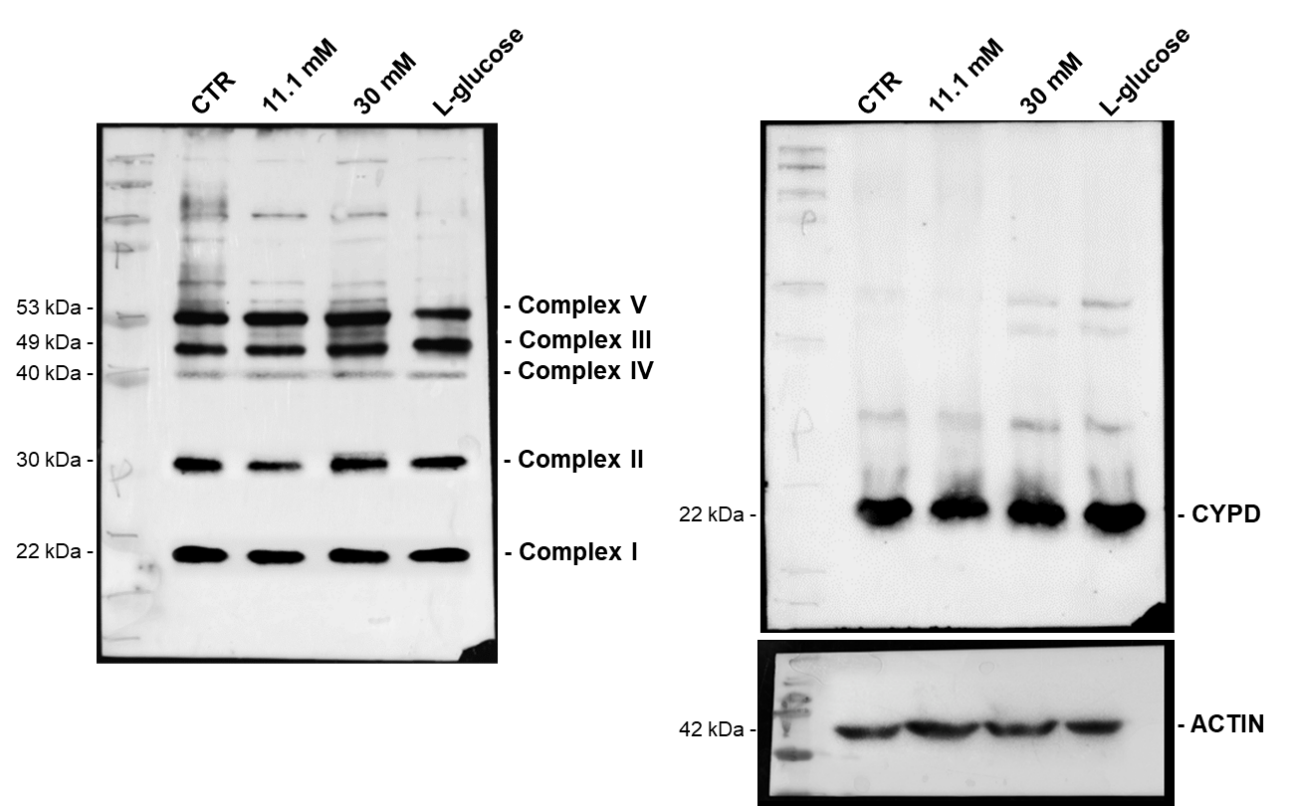
**FIGURE 4D – UNCROPPED:**

**Figure 4D – Uncropped**. Images of original uncropped Western blots used for preparation of Figure 4D. Western blot was performed on cell lysates using specific antibodies against OXPHOS complexes and CYPD. Actin was used as a marker of equal loading.


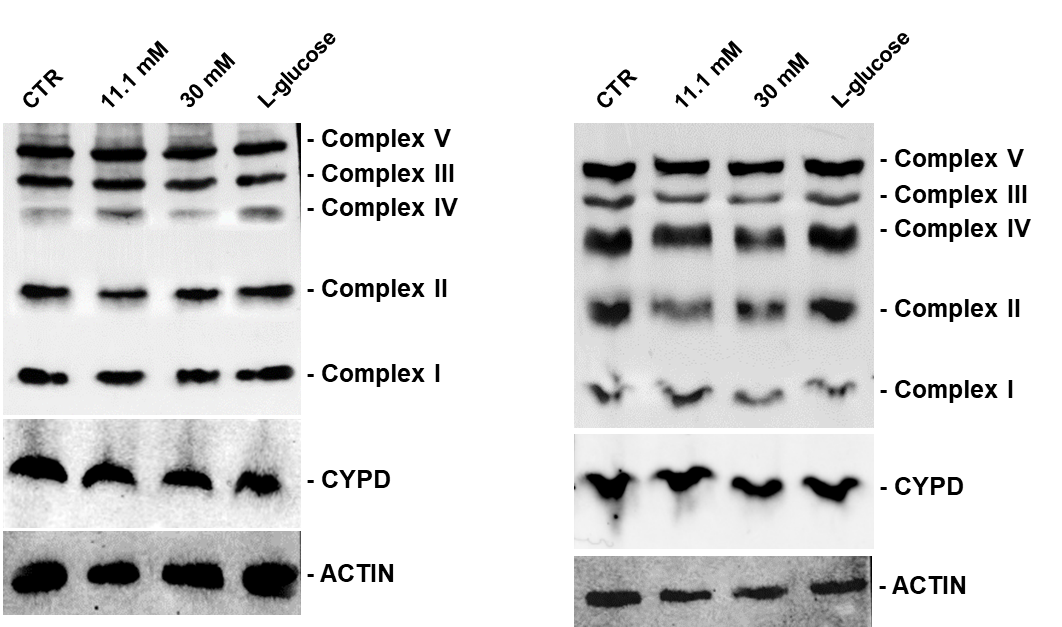
**FIGURE 4D – REPLICATES:**

**Figure 4D – Replicates**. Western blot replicates of Figure 4D.


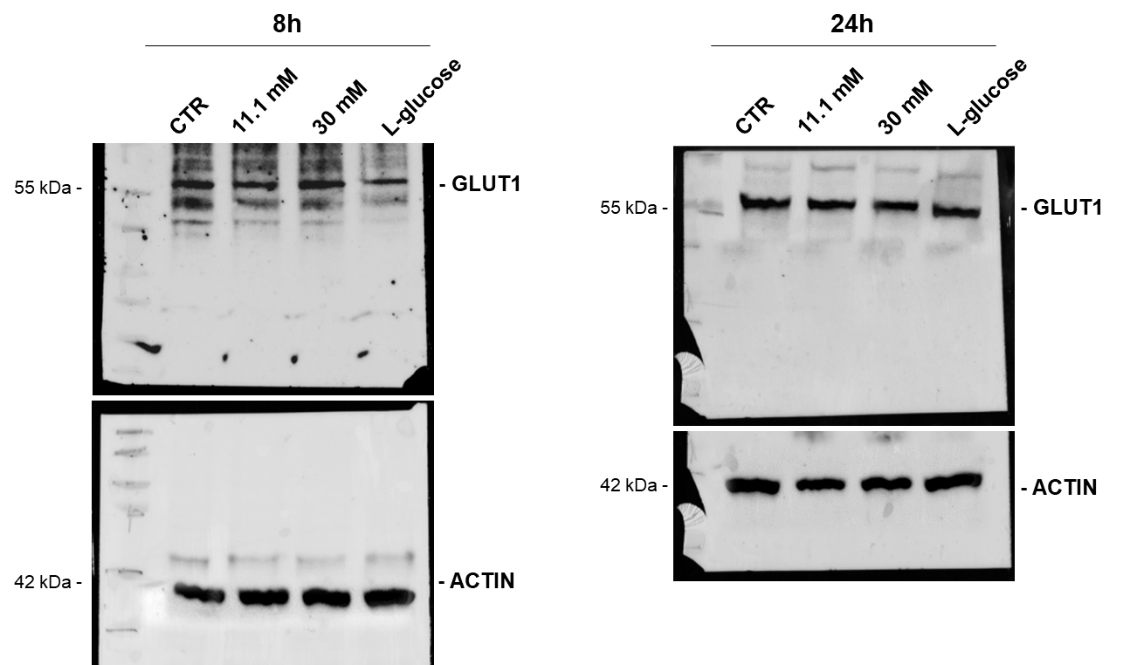
**FIGURE 5A – UNCROPPED:**

**Figure 5A – Uncropped**. Images of original uncropped Western blots used for preparation of Figure 4D. Western blot was performed on cell lysates using specific antibodies against GLUT1. Actin was used as a marker of loading.


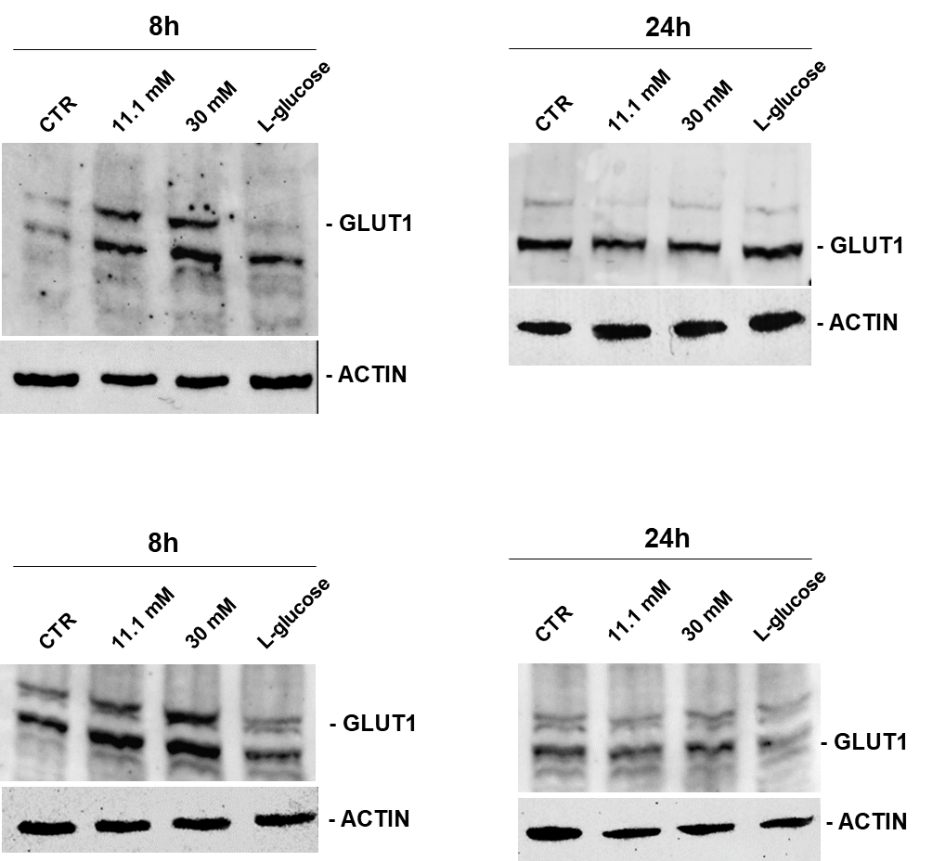
**FIGURE 5A – REPLICATES:**

**Figure 5A – Replicates**. Western blot replicates of Figure 5A.

**
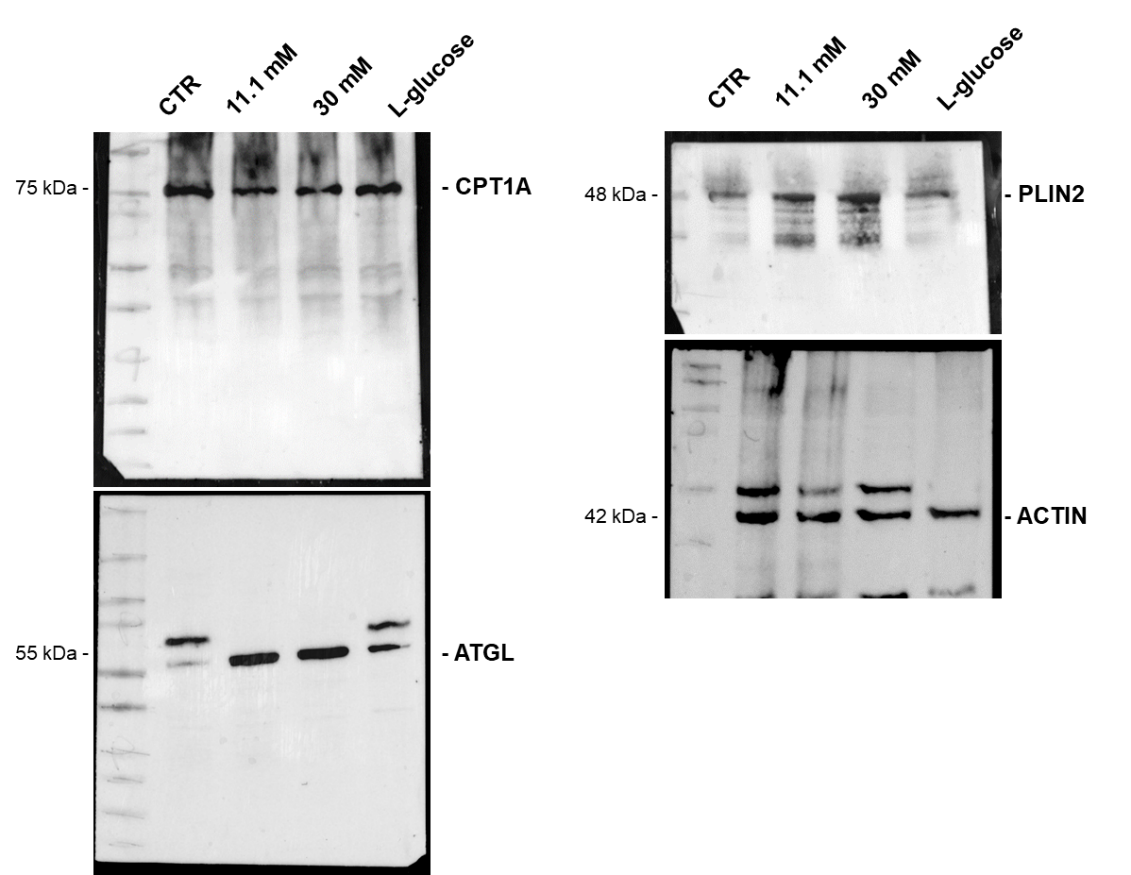
FIGURE 5C – UNCROPPED:**

**Figure 5C – Uncropped**. Images of original uncropped Western blots used for preparation of Figure 4D. Western blot was performed on cell lysates using specific antibodies against CTP1A, ATGL and PLIN2. Actin was used as a marker of loading.

**
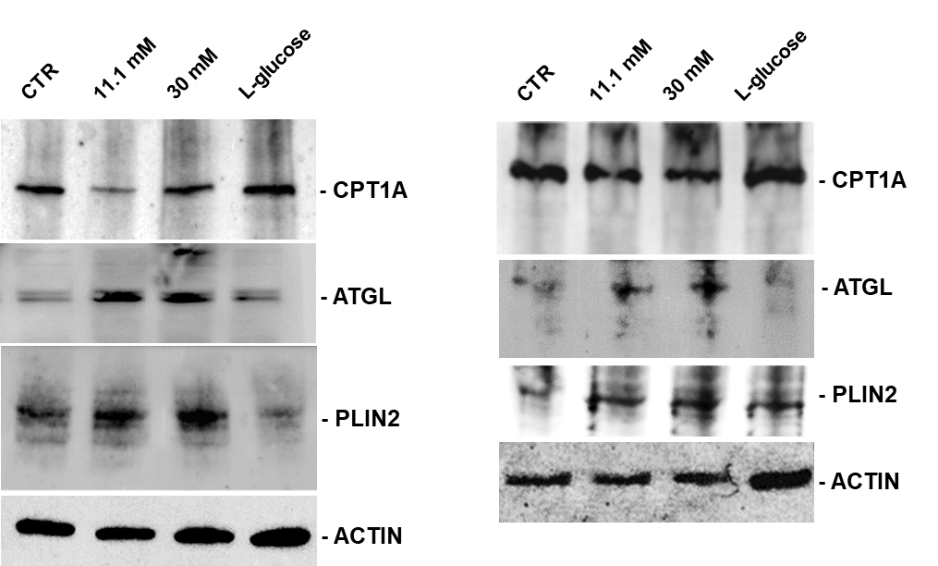
FIGURE 5C – REPLICATES:**

**Figure 5C – Replicates**. Western blot replicates of Figure 5C.
